# Anorexia nervosa adversity and emotion regulation: research and therapeutic perspectives

**DOI:** 10.1192/j.eurpsy.2026.10229

**Published:** 2026-07-31

**Authors:** M. Robin, H. Mayer-Benarous, P. Votadoro, L. Soukdeo, M. Corcos

**Affiliations:** 75014, Institut Mutualiste Montsouris, Paris, France

## Abstract

**Introduction:**

Whereas childhood adversity is a well-recognised risk factor in the development of psychiatric disorders, there is a lack of descriptive data in clinical population, especially in Eating Disorders (ED).

ED are characterized by difficulties in social communication, and most studies on emotion regulation have revealed disparities in ED samples. The sources of this heterogeneity remain to be explored, and adversity could play a role in determining distinct traumatic pathways, as it is the case for borderline personality disorders, a frequent comorbidity of ED.

Few studies described emotion regulation in adolescents, while delineating emotions perception profiles at this age could allow a better understanding of psychopathology and possibly orientate prevention and treatment.

**Objectives:**

This communication summarises the literature data on childhood adversity and on emotion regulation in ED. It then presents data from three original studies conducted on adversity and its links to emotional regulation in ED. The aim is to map psychopathological pathways between adversity factors and ED symptoms, to promote tailored therapeutic perspectives.

**Methods:**

In the FAMILY & CARE study, maltreatment and at-risk family interactions were measured in 425 hospitalized adolescents among psychiatric main disorders, including a subgroup of 92 adolescents with ED.

In the EURNET-BPD study, adolescents with borderline personality disorders (n = 85) were compared to healthy controls (n = 84), to measure emotional regulation characteristics (alexithymia, level of emotional awareness, facial affect recognition through a morphing paradigm, and empathy). An Eating Disorders sub-group (n = 47) was analysed as a comorbidity of borderline personality disorder.

In the RELADO study, 21 adolescents with ED were compared to 26 control patients and to 42 healthy adolescents. Experimental emotional and body perceptions were measured and correlated to adversity (abuse and neglect): alexithymia, interoceptive awareness, heart beat detection task.

**Results:**

Our results revealed that history of patients with ED was characterized by 39.6% of emotional abuse, 16.5% of physical abuse, 27.5% of sexual abuse and 74.7% of neglect. These patients were more accurate at detecting facial emotions than control patients, and showed higher emotional awareness of others. Their accuracy at detecting body signals (heartbeats) was higher than healthy controls, while self-emotional and interoceptive awareness was lower and correlated to body mass index, and to parental neglect.

These results were used to develop a trauma-informed therapeutic and preventive programme, Artemis, based on the physical and emotional perceptions in adolescents with ED.

**Conclusions:**

Our research confirms the importance of establishing links between adversity and emotional regulation in ED, in order to develop approaches that specifically target these psychopathological pathways.

**Disclosure of Interest:**

None Declared

